# Imaging tau burden in dementia with Lewy bodies using [^18^F]-AV1451 positron emission tomography

**DOI:** 10.1016/j.neurobiolaging.2020.11.006

**Published:** 2021-05

**Authors:** Elijah Mak, Nicolas Nicastro, Maura Malpetti, George Savulich, Ajenthan Surendranathan, Negin Holland, Luca Passamonti, P Simon Jones, Stephen F. Carter, Li Su, Young T. Hong, Tim D. Fryer, Guy B. Williams, Franklin Aigbirhio, James B. Rowe, John T. O'Brien

**Affiliations:** a Department of Psychiatry, School of Clinical Medicine, University of Cambridge, Cambridge, UK; b Department of Clinical Neurosciences, Geneva University Hospitals, Geneva, Switzerland; c Department of Clinical Neurosciences, School of Clinical Medicine, University of Cambridge, Cambridge, UK; d Wolfson Brain Imaging Centre, School of Clinical Medicine, University of Cambridge, Cambridge, UK

**Keywords:** Lewy bodies, Dementia, Tau, Amyloid, Neuroinflammation

## Abstract

Alzheimer's disease (AD) pathology is frequently observed as a comorbidity in people with dementia with Lewy bodies (DLB). Here, we evaluated the in vivo distribution of tau burden and its influence on the clinical phenotype of DLB. Tau deposition was quantified using [^18^F]-AV1451 positron emission tomography in people with DLB (n = 10), AD (n = 27), and healthy controls (n = 14). A subset of patients with Lewy body diseases (n = 4) also underwent [^11^C]-PK11195 positron emission tomography to estimate microglial activation. [^18^F]-AV1451 BP_ND_ was lower in DLB than AD across widespread regions. The medial temporal lobe [^18^F]-AV1451 BP_ND_ distinguished people with DLB from AD (AUC = 0.87), and negatively correlated with Addenbrooke's Cognitive Examination-Revised and Mini-Mental State Examination. There was a high degree of colocalization between [^18^F]-AV1451 and [^11^C]-PK11195 binding (*p* < 0.001). Our findings of minimal tau burden in DLB confirm previous studies. Nevertheless, the associations of [^18^F]-AV1451 binding with cognitive impairment suggest that tau may interact synergistically with other pathologic processes to aggravate disease severity in DLB.

## Highlights

• We evaluated [18F]-AV1451 uptake in dementia with Lewy bodies (DLB) and Alzheimer's disease (AD).
• There is minimal tau deposition in DLB compared to healthy controls.
• Tau imaging may be useful for differential diagnosis of DLB and AD.
• Tau deposition was correlated with cognitive impairment in DLB.

## 1 Introduction

Dementia with Lewy bodies (DLB) is a common cause of dementia. It is pathologically defined by the presence of Lewy bodies but is often associated with coexisting Alzheimer's disease (AD) with variable degrees of amyloid plaques and neurofibrillary tangles (NFTs) (Nedelska et al., 2015; Schneider et al., 2007) in 50%–80% of cases in autopsy series (Irwin et al., 2017; Marui et al., 2004). This pathologic overlap complicates the differential diagnosis of AD versus DLB. Evidence of beta-amyloid (Aβ) is frequently observed in positron emission tomography (PET) studies of patients with probable DLB (Donaghy et al., 2015; Edison et al., 2008; Gomperts et al., 2008; Kantarci et al., 2012). The spatial progression of Aβ in DLB is consistent with that of AD and is associated with medial temporal lobe atrophy (Mak et al., 2019; Sarro et al., 2016) and cognitive impairment (Gomperts et al., 2012).

Postmortem data and cerebrospinal fluid (CSF) studies have demonstrated the influence of comorbid tau on clinical heterogeneity, brain imaging, and survival rates (Coughlin et al., 2019a,b; Howlett et al., 2015; Irwin et al., 2017; Nelson et al., 2012). For instance, DLB patients with low Braak stages had more frequent visual hallucinations (Merdes et al., 2003) and severe visual impairment (Peavy et al., 2016) relative to those with higher Braak stages. Beyond its influence on clinical and cognitive characteristics in DLB, concomitant tau burden was also associated with more severe medial temporal lobe atrophy (Burton et al., 2012, 2009), which is in keeping with longitudinal data showing accelerated rates of brain atrophy among a subset of DLB cases with substantial tau pathology (Nedelska et al., 2015).

Taken together, these studies have led to a growing recognition of the potentiating role of tau in the disease progression of DLB, as reflected by its integration into the neuropathologic assessment of DLB (McKeith et al., 2017). However, the contributions of in vivo tau deposition to the clinical manifestations and disease course of DLB remain unclear (Hall et al., 2017). Two of the first studies in DLB have reported greater [^18^F]-AV1451 uptake in the inferior temporal gyrus, precuneus, and occipital regions than healthy controls (Gomperts et al., 2016; Kantarci et al., 2016). Consistent with the structural preservation of medial temporal lobe (Firbank et al., 2010; Mak et al., 2017), Kantarci et al. further demonstrated that [^18^F]-AV1451 uptake in the medial temporal lobe led to a compelling separation between DLB and AD, highlighting the potential utility for differential diagnosis with tau imaging. These studies have because been validated by convergent findings, and there is a general consensus that tau burden in DLB is intermediate between healthy controls and AD, although the level of concomitant amyloid may be highly influential (Coughlin et al., 2020; Lee et al., 2018; Smith et al., 2018). Perhaps owing to the clinical heterogeneity of DLB (Walker et al., 2012), mixed findings have been reported on the relationships between [^18^F]-AV1451 and cognition (Coughlin et al., 2020; Gomperts et al., 2016; Kantarci et al., 2016). Moreover, overt cognitive impairment may arise through the interplay among tau, alpha-synuclein, and Aβ (Howlett et al., 2015) and neuroinflammation (Mackenzie, 2000; Surendranathan et al., 2018, 2015) (Sheffield et al., 2000).

Clarifying the contributions of tau to the pathophysiology of DLB is a key requirement to determine whether emerging antitau interventions may also have therapeutic potential in patients with DLB. This study had three objectives: (i) to compare the spatial distribution of [^18^F]-AV1451 binding in DLB against HC and AD; We predicted that [^18^F]-AV1451 binding would be substantially lower in DLB than AD, distinguishing DLB from AD with high accuracy; (ii) to determine the clinical relevance of [^18^F]-AV1451 binding in DLB by delineating its associations with cognitive performance; and (iii) to assess the topographical relationship between tau ([^18^F]-AV1451 binding) and neuroinflammation ([^11^C]-PK11195 binding (Mak et al., 2018; Surendranathan et al., 2018, 2015) given the synergy between microglial activation and tau pathology (See Vogels et al., 2019 for a review).

## 2 Materials and methods

### 2.1 Participants and clinical data

The data are from the Neuroimaging of Inflammation in MemoRy and Related Other Disorders (Bevan-Jones et al., 2017) and tau Evaluation and Neurodegeneration in Dementia Research protocols. We include 51 participants (10 DLB, 14 HC, 14 amyloid-positive patients with mild cognitive impairment (MCI), and 13 early AD) above age 50 years. Patients were recruited from specialist memory clinics in and around Cambridgeshire, the Dementias and Neurodegeneration specialty of the UK Clinical Research Network (DeNDRoN) or the Join Dementia Research platform (www.joindementiaresearch.nihr.ac.uk). Probable DLB was defined by both 2005 and 2017 consensus criteria, and all patients who met the 2005 criteria also met the 2017 criteria (McKeith et al., 2017, 2005). 3 of the DLB patients underwent ^123^I-FP-CIT imaging, all of which were rated as abnormal MCI was defined as Mini-Mental State Examination (MMSE) > 24, with memory impairments beyond what is expected for age and education and not explained by another diagnosis (Albert et al., 2011). MCI patients were all amyloid-positive, defined by an average cortical standardized uptake value ratio (SUVR) > 1.5 for Pittsburgh Compound-B ([^11^C]-PiB) PET (Villemagne et al., 2013). Probable AD was defined as per the National Institute on Aging-Alzheimer's Association guidelines (McKhann et al., 2011). AD and amyloid-positive MCI participants were combined into a single group, representing a continuum of AD severity (Okello et al., 2009). Healthy controls were recruited from the Join Dementia Research register and local registers. Healthy controls had MMSE >26, with no cognitive symptoms or unstable/significant medical illness. Participants did not have any acute infectious or chronic symptomatic systemic inflammatory disorder (e.g., systemic lupus erythematosus and rheumatoid arthritis) or contraindications on magnetic resonance imaging (MRI). Global cognition was assessed using the MMSE and Addenbrooke's Cognitive Examination-Revised (ACE-R). The study was approved by the East of England Ethics Committee (13/EE/0104; 16/EE/0529) and the UK Administration of Radioactive Substances Advisory Committee.

### 2.2 Clinical assessments

Participants underwent an initial assessment that included neuropsychological and cognitive testing, including MMSE and ACE-R, severity of parkinsonism (Unified Parkinson's Disease Rating Scale part III—motor (Goetz et al., 2008) and demographic measures.

### 2.3 Structural MRI

All neuroimaging was conducted on the Cambridge Biomedical Campus, Cambridge, UK. Participants underwent an MRI session acquired on a 3T scanner (Siemens Magnetom Tim Trio and Verio scanners; Siemens Healthineers, Erlangen, Germany) using a magnetization-prepared rapid gradien echo T1-weighted sequence. The T1-weighted sequence (repetition time = 2300 ms, echo = 2.98 ms, field of view = 240 × 256 mm, 176 slices of 1 mm thickness, flip angle = 9°) was used to facilitate tissue class segmentation (gray and white matter, together with CSF) and to allow nonrigid registration of standard space regions of interest (ROIs) to subject MRI space. Each T1-weighted image was nonrigidly registered to the ICBM2009a template brain using ANTS (http://www.picsl.upenn.edu/ANTS/), and the inverse transform was applied to the standard space ROIs (a modified version of the n30r87 Hammersmith atlas (www.brain-development.org) resliced from MNI152 to ICBM2009a space that included the midbrain and dentate nucleus of the cerebellum) to bring the regions of interest to subject MRI space.

### 2.4 PET imaging of [^18^F]-AV1451, [^11^C]-PiB and [^11^C]-PK11195

All participants underwent [^18^F]-AV1451 PET to assess the extent and intensity of brain tau pathology. All MCI and 5 patients with DLB also underwent [^11^C]-PiB PET to assess the density of Aβ deposits as an indication of AD pathology. For our proof-of-concept analysis to study the topographical association between tau and microglial activation, 3 patients with DLB and 1 additional patient with Parkinson's disease with dementia had both [^18^F]-AV1451 and [^11^C]-PK11195 PET. All radioligands were prepared at the Wolfson Brain Imaging Center (WBIC), University of Cambridge, with high radiochemical purity (>95%). [^18^F]-AV1451–specific activity was 216 ± 60 GBq/μmol at the end of synthesis, whereas [^11^C]-PiB was produced with specific activity >150 GBq/μmol. PET scanning was performed on a GE Advance PET scanner and a GE Discovery 690 PET/CT (GE Healthcare, Waukesha, USA). A 15-min ^68^Ge/^68^ Ga transmission scan was used for attenuation correction on the Advance, which was replaced by a low-dose CT scan on the Discovery 690. The emission protocols were the same on both scanners: 90 minutes dynamic imaging after a 370 MBq [^18^F]-AV1451 injection, 40–70 minutes postinjection acquisition after a 550 MBq [^11^C]-PiB injection and 75 minutes dynamic imaging after a 500 MBq [^11^C]PK11195 injection.

For all tracers, each emission frame was reconstructed using the PROMIS 3D filtered back projection algorithm (Kinahan and Rogers, 1989) into a 30 cm transaxial field of view (128 × 128 matrix), with a transaxial Hann filter cutoff at the Nyquist frequency. Corrections were applied for randoms, dead time, normalization, scatter, attenuation, and sensitivity. Each emission image series was aligned using SPM12 (www.fil.ion.ucl.ac.uk/spm/software/spm12) to ameliorate the effect of patient motion during data acquisition. The mean aligned PET image, and hence the corresponding aligned dynamic PET image series, was rigidly registered to the T_1_-weighted image using SPM12 to extract radioactivity concentration values from both the Hammersmith atlas ROIs and the reference region.

For both [^18^F]-AV1451 and [^11^C]-PiB, the reference region was defined in cerebellar gray matter using a 90% lower threshold on the gray matter probability map produced by SPM12 smoothed to PET spatial resolution. [^18^F]-AV1451 results were obtained with and without correction for CSF partial volume error. For the CSF corrected data, all ROI time-activity curves, including that for the reference tissue, were corrected through division with the ROI gray matter plus white matter tissue fraction obtained from SPM12 probability maps smoothed to PET spatial resolution. For ^18^F-AV-1451 nondisplaceable binding potential (BP_ND_), a measure of specific binding was determined for each Hammersmith atlas ROI using a basis function implementation of the simplified reference tissue model (Gunn et al., 1997). [^11^C]-PiB data were quantified using SUVR by dividing the mean radioactivity concentration in cortical ROIs by the corresponding mean radioactivity concentration in the reference tissue ROI. [^11^C]-PiB data were treated as dichotomous measures (i.e., positive or negative) and considered positive if the volume-weighted average SUVR value across the cortical ROIs was >1.5 (Villemagne et al., 2013). For [^11^C]-PK11195 the reference tissue, the TAC was determined from supervised cluster analysis, and BP_ND_ was estimated using a simplified reference tissue model algorithm incorporating vascular binding correction (Yaqub et al., 2012). [^11^C]-PK11195 ROI time-activity curves were CSF corrected as described above for [^18^F]-AV1451, whereas the [^11^C]-PK11195 reference tissue TAC was CSF corrected through voxel-wise correction using the smoothed CSF probability map before the supervised cluster analysis. Outputs of the multiple steps used to produce the ROI [^18^F]-AV1451 BP_ND_—image reconstruction, image realignment, coregistration of PET and MRI, reference tissue ROI delineation and TAC, spatial normalization for target ROI delineation, kinetic model fits—were all assessed by experienced PET analysts (YTH and TDF). All partial volume–corrected PET data were subsequently used for statistical analyses. The agreement between non-partial volume corrected and partial volume corrected datasets are illustrated in the Supplementary Material.

### 2.5 Statistical analyses

Demographic variables were compared between the groups using t-tests, Wilcoxon rank sum tests, chi-square tests, and analysis of covariance or Kruskal-Wallis tests where appropriate based on normality tests. Owing to the skewed distributions, regional [^18^F]-AV1451 BP_ND_ were subjected to inverse normal transformations, so as to fulfill the normality assumptions of linear regression and ANOVA (Ganjgahi et al., 2015; Hodgson et al., 2017; Raffield et al., 2015; Tynkkynen et al., 2018). Group-wise differences between whole-brain [^18^F]-AV1451 BP_ND_ were evaluated with ANCOVA and post hoc Tukey tests, adjusted with false discovery rate (FDR) for multiple comparisons. We used area under the receiver operating characteristic curves (AUC) to assess the accuracy of (i) medial temporal lobe [^18^F]-AV1451 BP_ND_ to distinguish between DLB and AD. Linear regression models, adjusted for age, were used to assess the relationship between regional [^18^F]-AV1451 BP_ND_ with cognitive variables such as ACE-R and MMSE. Topographical relationships between [^18^F]-AV1451 and [^11^C]-PK11195 binding were assessed using linear mixed effects models, adjusting for the fixed effects of age, as well as the random factor of subject to account for the repeated measurements of ROIs.

### 2.6 Data availability

Data are available upon reasonable requests.

## 3 Results

### 3.1 Sample characteristics

The demographics and clinical variables are summarized in Table 1. ACE-R and MMSE scores were lower in AD and DLB relative to controls (*p* < 0.001), but there were no differences between DLB and AD. Compared with the controls, the DLBs were older and had lower education years. Of the 5 patients with DLB with [^11^C]-PiB scans, 2 were positive and 3 were negative.

**Table 1:** Participant characteristics

Variables	HC (N = 14)	DLB (N = 10)	AD (N = 27)	*p*-value
Age				
Mean (SD)	67.4 (8.21)	78.3 (6.00)	72.5 (8.83)	0.009
Median [min, max]	69.0 [55.0, 80.0]	77.0 [71.0, 86.0]	75.0 [53.0, 86.0]	
Gender				
Female	7 (50.0%)	2 (20.0%)	12 (44.4%)	0.298
Male	7 (50.0%)	9 (80.0%)	15 (55.6%)	
Edu				
Mean (SD)	15.8 (1.93)	11.7 (2.58)	13.1 (3.12)	0.003
Median [min, max]	16.0 [11.0, 19.0]	10.5 [9.00, 17.0]	12.0 [10.0, 19.0]	
ACER				
Mean (SD)	93.6 (4.69)	73.6 (19.6)	77.5 (9.06)	<0.001
Median [min, max]	95.0 [82.0, 99.0]	76 [36.0, 96.0]	80.0 [53.0, 91.0]	
MMSE				
Mean (SD)	29.1 (0.829)	23.5 (5.15)	25.4 (2.32)	<0.001
Median [min, max]	29.0 [28.0, 30.0]	25.5 [12.0, 28.0]	26.0 [19.0, 28.0]	
UPDRS				
Mean (SD)		25.0 (11.7)		NA
Median [min, max]		26.0 [11.0, 52.0]		

Key: ACE-R, Addenbrooke's Cognitive Examination-Revised; AD, Alzheimer's disease; DLB, Dementia with Lewy bodies; HC, Healthy controls; MMSE, Mini-Mental State Examination.

### 3.2 Comparisons of [^18^F]-AV1451 binding

**Fig. 1:**
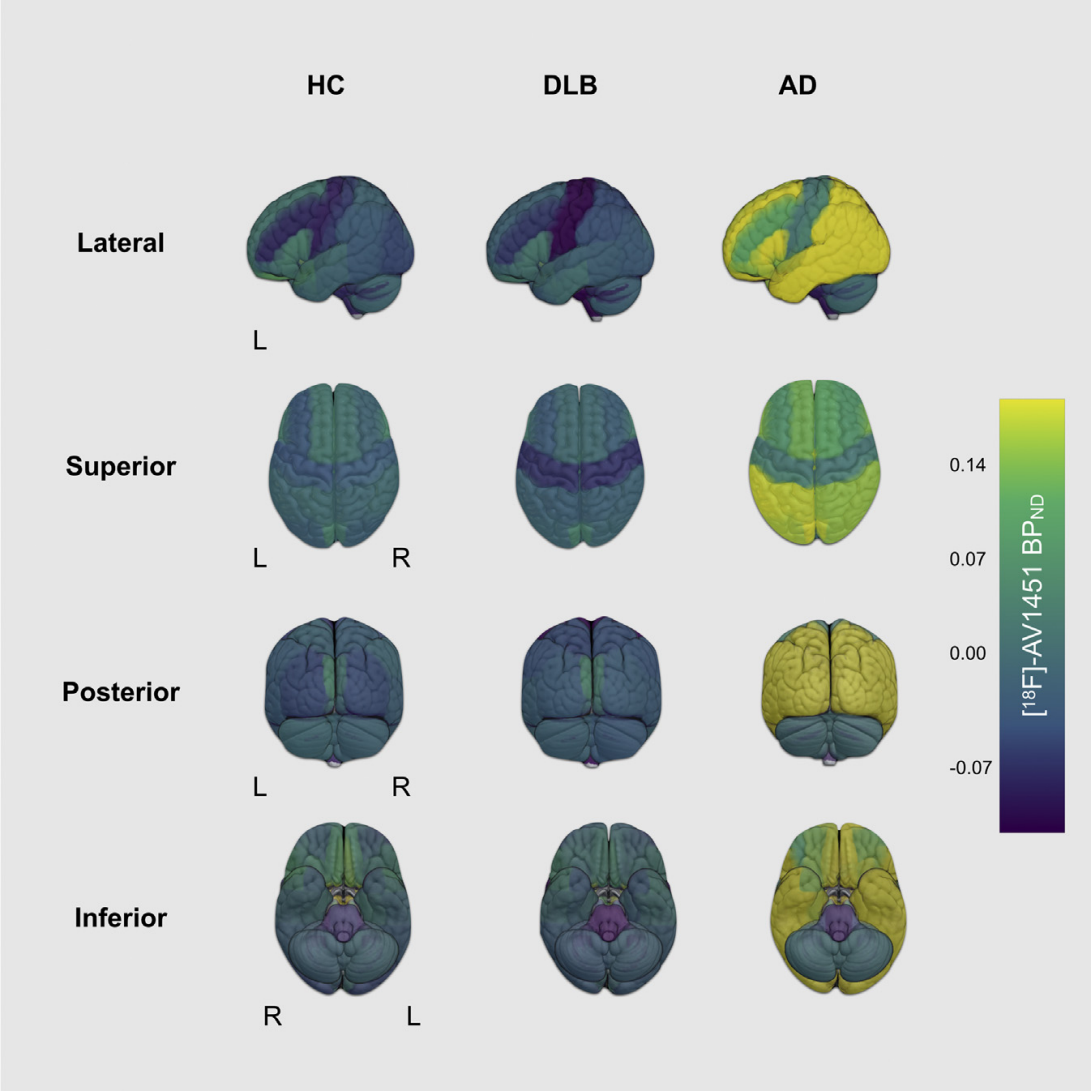
Regional distribution of [^18^F]-AV1451 binding (BP_ND_) in DLB, HC, and AD. Abbreviations: AD, Alzheimer's disease; DLB, dementia with Lewy bodies; HC, healthy controls; L, left hemisphere; R, right hemisphere.

Group averaged [^18^F]-AV1451 BP_ND_ for each ROI is overlaid on the Hammersmith atlas in Fig. 1 and boxplots are depicted in Supplementary Fig. 1. There were no significant differences between regional [^18^F]-AV1451 BP_ND_ for healthy controls and DLB (FDR *p* > 0.05). A widespread pattern of increased [^18^F]-AV1451 binding was observed in AD compared with DLB (FDR *p* < 0.05) (Supplementary Fig. 2); predominantly found across the temporal, parietal, and frontal cortices, as well as the cingulate and subcortical regions including the left hippocampus; bilateral amygdala; and nucleus accumbens, thalamus, right pallidum, and right putamen. No regions showed higher [^18^F]-AV1451 BP_ND_ in DLB relative to AD.

### 3.3 Medial temporal lobe [^18^F]-AV1451 binding

**Fig. 2:**
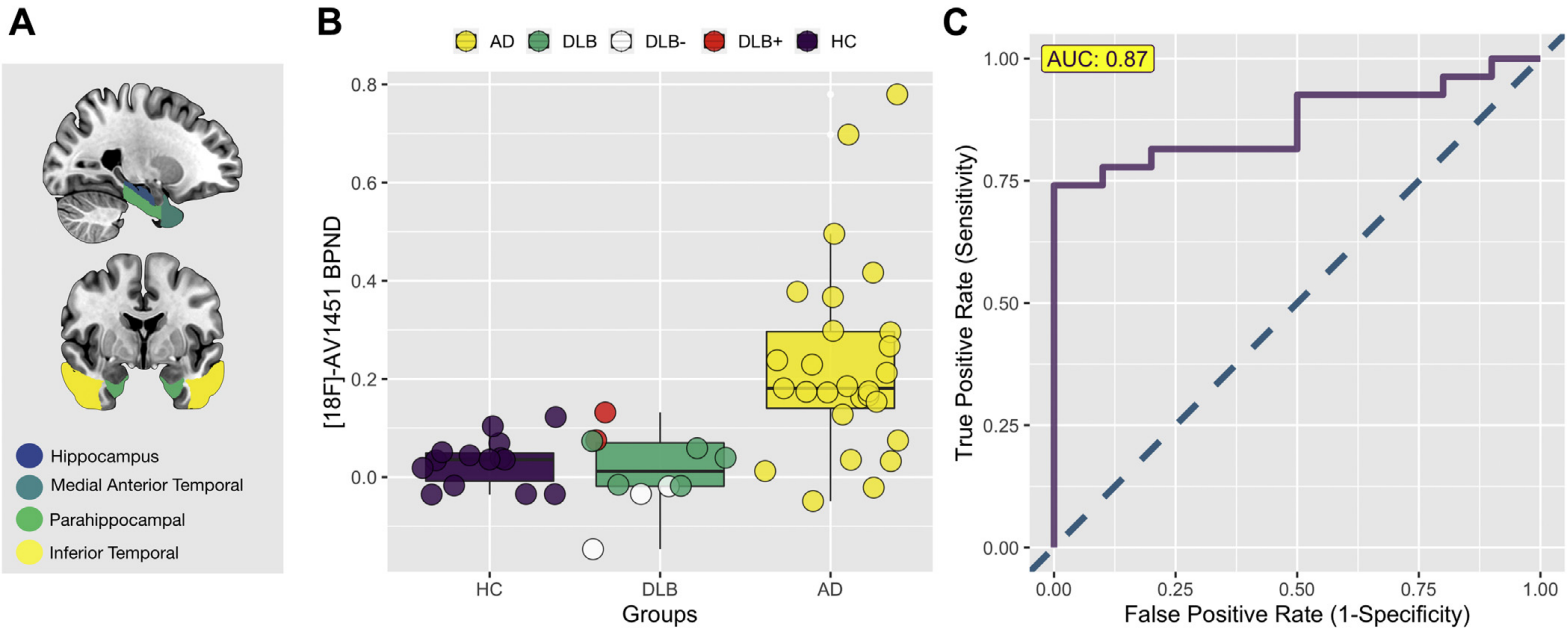
[^18^F]-AV1451 binding (BP_ND_) in the medial temporal lobe between DLB and AD. (A) Meta-ROI of the medial temporal lobe. (B) Group differences in [^18^F]-AV1451 binding. (C) AUC analyses distinguishing DLB from AD. Abbreviations: AD, Alzheimer's disease; AUC = area under the receiver operating characteristic curves; DLB, dementia with Lewy bodies; HC, healthy controls; MTL, medial temporal lobe; ROI, region of interest.

Because elevations of [^18^F]-AV1451 binding have been consistently observed in medial temporal regions in AD cases (Coughlin et al., 2020; Kantarci et al., 2016), we constructed a medial temporal lobe meta-ROI composed of the hippocampus, medial anterior temporal lobe, parahippocampal gyrus, and the inferior temporal lobe ROIs to investigate the discriminatory value of medial temporal lobe [^18^F]-AV1451 retention to differentiate DLB from AD (Fig. 2A). [^18^F]-AV1451 BP_ND_ of the medial temporal lobe was significantly elevated in AD compared with DLB (F = 10.1, *p* = 0.003, adjusted for age). Moreover, our receiver operating characteristic analyses showed that the medial temporal lobe [^18^F]-AV1451 BP_ND_ distinguished DLB and AD with an AUC of 0.87 (Fig. 2). For visualization purposes, the DLB Aβ+ (n = 2) and DLB Aβ- (n = 3) are color coded as red and white, respectively.

### 3.4 Cognitive correlates of [^18^F]-AV1451 binding in DLB

**Fig. 3:**
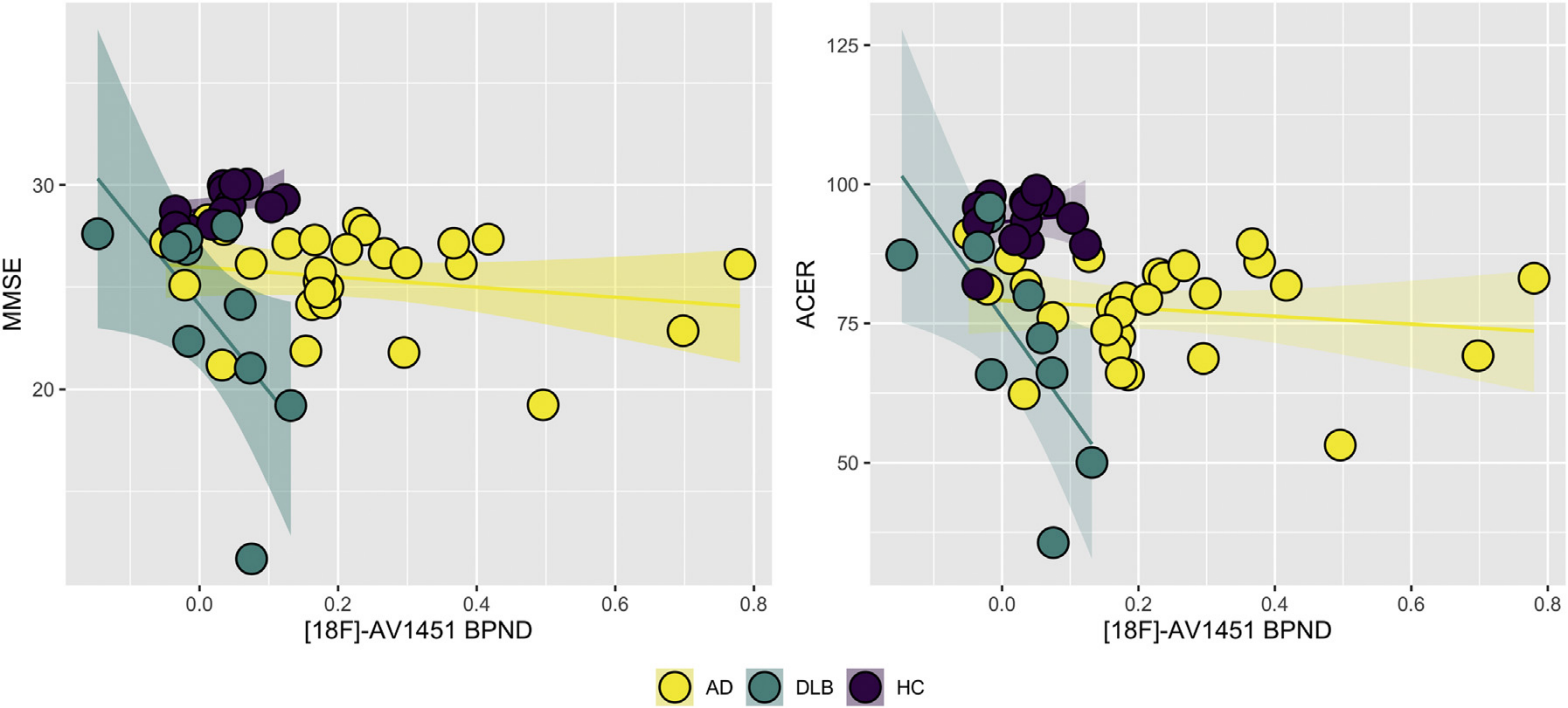
[^18^F]-AV1451 binding (BP_ND_) in the medial temporal lobe is associated with cognitive impairment in DLB. Abbreviations: ACE-R, Addenbrooke's Cognitive Examination Revised; AD, Alzheimer's disease; DLB, dementia with Lewy bodies; HC, healthy controls, MMSE, Mini-Mental State Examination.

Scatter plots of medial temporal lobe [^18^F]-AV1451 BP_ND_ against ACE-R and MMSE are shown in Fig. 3. Within the DLB group, medial temporal lobe [^18^F]-AV1451 BP_ND_ was significantly correlated with both ACE-R and MMSE scores after adjusting for age (Spearman R = -0.8 for ACER and MMSE, *p* < 0.01). There was a significant [^18^F]-AV1451 BP_ND_ × group interaction with ACE-R and MMSE, such that the association between higher medial temporal lobe [^18^F]-AV1451 BP_ND_ and lower ACE-R and MMSE scores were exclusive to the DLB group (*p* < 0.05).

### 3.5 Colocalization of tau and neuroinflammation

**Fig. 4:**
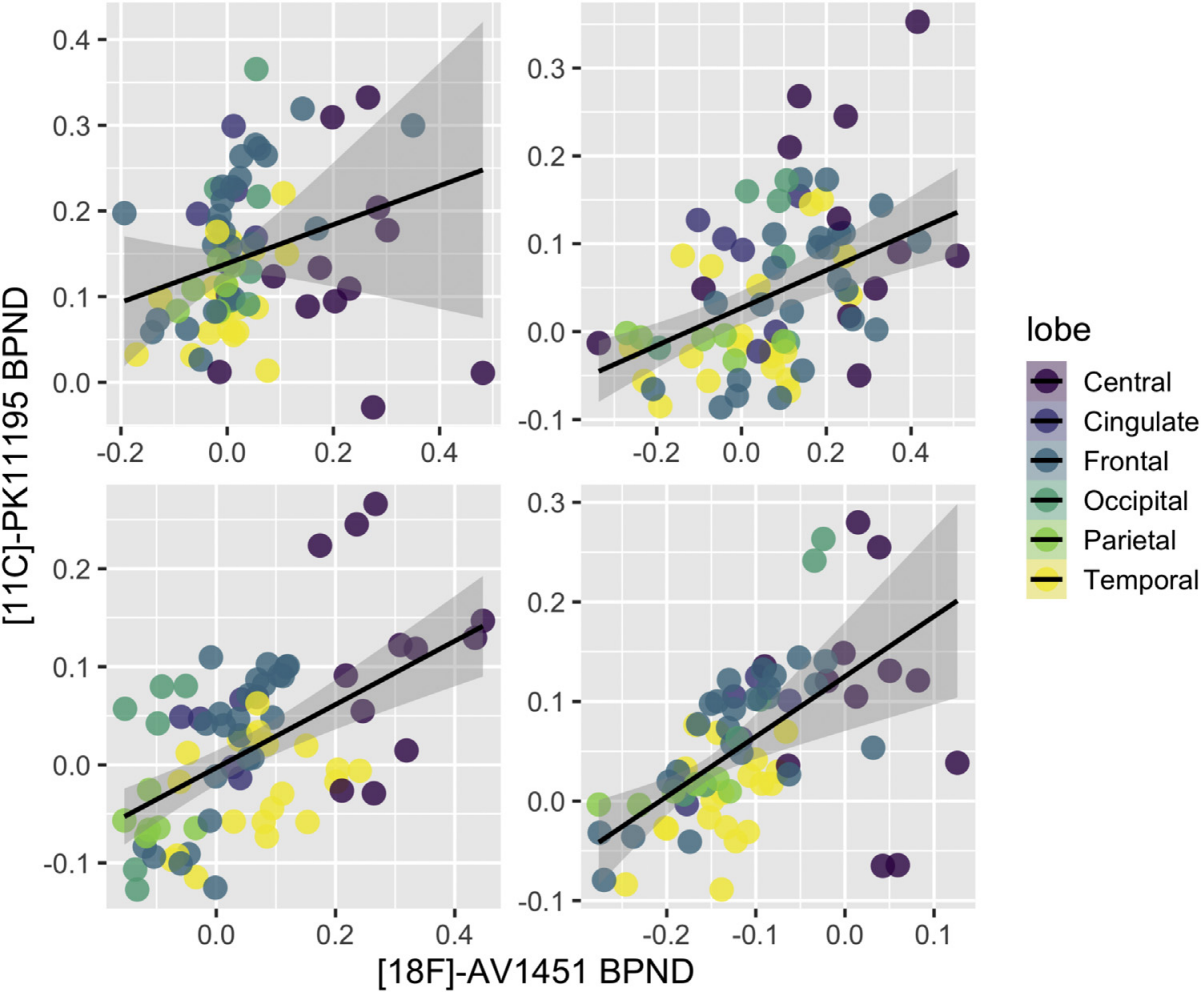
Colocalization of [^18^F]-AV1451 and [^11^C]-PK11195 BP_ND_ in each of the 4 patients with LBD who had scans with both tracers. Abbreviations: LBD, Lewy body disorders, ∗ Patient with PDD.

In this proof-of-concept substudy, we showed scatter plots of regional [^18^F]-AV1451 and [^11^C]-PK11195 BP_ND_ for each of our patients with Lewy body diseases (3 DLB and 1 Parkinson's disease with dementia) with both tracers (Fig. 4). Linear mixed effects models (adjusted for age and subject as random factor) indicated a strong topographical relationship between [^18^F]-AV1451 and [^11^C]-PK11195 BP_ND_ across the subjects (beta = 0.66, SE = 0.08, 95% CI [0.05, 0.08], t = 7.97, *p* < 0.001), suggesting a similar pattern of accrual for both tau and neuroinflammation across the brain. This group-level linear mixed effect finding was further supported by separate robust linear regressions, showing significant associations between both tracers within each subject (2.8 < T < 8.8, *p* < 0.05).

## 4 Discussion

The principal result of this study is that [^18^F]-AV1451 binding is not elevated in DLB relative to normal aging. This is in partial agreement with previous in vivo studies (Gomperts et al., 2016; Kantarci et al., 2016; Smith et al., 2018) and neuropathologic reports (Jellinger and Attems, 2008). In addition, our findings confirmed (i) the high discriminative performance of tau imaging to distinguish DLB from AD (Kantarci et al., 2016; Ossenkoppele et al., 2018) and (ii) support a role of tau to underlie cognitive dysfunction in DLB (Gomperts et al., 2016).

Elevated [^18^F]-AV1451 uptake in the temporal, parietal, and occipital cortices has been described in DLB (Gomperts et al., 2016; Kantarci et al., 2016). However, the reported effect sizes have been modest, and the spatial distribution of [^18^F]-AV1451 binding inconsistent across occipital (Kantarci et al., 2016), temporal (Gomperts et al., 2016), and parietal cortex (Smith et al., 2018). There are several plausible explanations for the comparable [^18^F]-AV1451 binding levels seen in our DLB and controls. First, previous studies have explicitly included controls who were Aβ-negative (Coughlin et al., 2020; Gomperts et al., 2016; Kantarci et al., 2016). Elevated cortical Aβ occurs in cognitively normal elderly (Chetelat et al., 2013), along with tau accumulation within the temporal cortex (Johnson et al., 2016). In our study, [^11^C]-PiB PET imaging was not available in controls, and thus we cannot discount the possibility that latent Aβ may have obscured group differences in [^18^F]-AV1451 binding between DLB and controls. This is an important consideration because Aβ-associated tau accumulation is a frequent characteristic of normal aging with cognitive implications (Schöll et al., 2016; Ziontz et al., 2019). Similarly, the unavailability of [^11^C]-PiB PET imaging in 5 of our 10 patients with DLB precluded group stratification by Aβ status. Indeed, recent evidence indicated that [^18^F]-AV1451 elevations in DLB may be dependent on Aβ status (Coughlin et al., 2020). In that study, higher total mean cortical tau was demonstrated in the subgroup of patients with LBD with greater Aβ burden, whereas the difference between the Aβ-negative LBD patients and controls only trended toward statistical significance. Taken together, our data are not necessarily at odds with previous reports of focal [^18^F]-AV1451 elevations in DLB relative to controls, (Coughlin et al., 2020; Gomperts et al., 2016; Kantarci et al., 2016), because these findings are—to a large extent—likely to be driven by a subset of DLB cases who also have concomitant amyloid accumulation (Coughlin et al., 2020). The minimal levels of [^18^F]-AV1451 burden in our DLB sample may also be related to the mild severity of DLB in our cohort (mean MMSE of 24). This hypothesis lends itself to be challenged with further longitudinal studies involving a series of people with moderate to severe DLB.

As predicted, the AD group showed widespread elevated [^18^F]-AV1451 binding compared with DLB. In particular, [^18^F]-AV1451 BP_ND_ in the medial temporal lobe discriminated DLB from AD with an AUC of 0.87, closely mirroring previous findings from Kantarci et al., (2016) (AUC = 0.99) and Ossenkoppele et al. (2018) (AUC = 0.88) (Kantarci et al., 2016; Ossenkoppele et al., 2018). While the range of AUCs exceed the conventional threshold to be considered as clinically meaningful (Fan et al., 2005), it is still unclear whether the binding affinity of the [^18^F]-AV1451 tracer may be compromised due to the lack of hyperphosphorylated tau in DLB. Lending credence to this notion, a previous study used digital quantification of tau deposition and found much higher degrees of tau density in AD to DLB that were disproportionate to the Braak tau stages (Coughlin et al., 2019a,b).

In the context of differential diagnosis between DLB and AD, the discriminative ability of medial temporal lobe [^18^F]-AV1451 BP_ND_ as shown in our study and by other research groups may lay the foundation for future efforts to validate the use [^18^F]-AV1451 PET in improving the differential diagnosis of Lewy body diseases, atypical parkinsonisms, and AD. Considering the similar AUCs achieved by both the medial temporal lobe [^18^F]-AV1451 uptake (Kantarci et al., 2016; Ossenkoppele et al., 2018) and ^123^I-FP-CIT SPECT (Maltais et al., 2020; Nicastro et al., 2017; O'Brien et al., 2014; Thomas et al., 2017) in the differentiation of DLB from AD, future efforts are warranted to evaluate whether the combination of [^18^F]-AV1451 PET and ^123^I-FP-CIT SPECT would lead to better accuracy in differential diagnosis relative to each modality alone. ^123^IFP-CIT-SPECT is not sensitive to cortical pathology. Therefore, one scenario in which the use medial temporal lobe [^18^F]-AV1451 uptake may be particularly helpful is in rare cases of false-negatives from ^123^I-FP-CIT SPECT scans; i.e. in DLB patients where nigrostriatal degeneration is minimal, and cortical pathology is the prominent feature.

The correlation between tau burden and cognitive decline may also suggest a therapeutic potential for antitau interventions in patients with DLB. Here, the significant associations between medial temporal lobe [^18^F]-AV1451 binding and worse global cognitive function lends support to a prior report in a mixed LBD sample (Coughlin et al., 2020; Gomperts et al., 2016). The clinical relevance of this correlation was also reinforced by a significant group × [^18^F]-AV1451 binding interaction, indicating that tau was more strongly related to poorer cognition in the DLB group relative to healthy controls or AD. Collectively, these findings and others (Coughlin et al., 2020; Gomperts et al., 2016) suggest a potential underlying role of tau in the disease course of DLB and warrant further longitudinal studies, ideally enrolling prodromal DLB patients to better understand if and how tau burden contributes to the disease course of Lewy body diseases. Nevertheless, how tau pathology may underlie cognitive impairment in DLB is still unclear. Given the low levels of [^18^F]-AV1451 binding in our study, tau accrual itself is unlikely to be the dominant driver of disease severity in patients with DLB. For instance, cognitive impairment could be exacerbated through the interaction of NFTs with other pathologic factors such as inflammation. Neuroinflammation is increasingly recognized as an important component in the disease course of DLB (Surendranathan et al., 2015) and mounting evidence suggests that both pathologies are intricately associated (Bhaskar et al., 2010; Vogels et al., 2019). In a proof of concept substudy, we found a high degree of colocalization between [^18^F]-AV1451 and [^11^C]-PK11195 binding in each of the patients with LBD who underwent scans with both tracers. Notwithstanding the need for further confirmation in larger independent samples, this preliminary finding is consistent with our previous work across the spectrum of frontotemporal dementia (Bevan-Jones et al., 2020), and together mirror the tendency for activated microglia to coalesce around NFT-bearing neurons (Sheffield et al., 2000), which in turn interact synergistically with Aβ (Gomperts et al., 2008) and alpha-synuclein (Colom-Cadena et al., 2013; Howlett et al., 2015). Future studies that are designed to disentangle the associations across neuroinflammation, Aβ, tau, and alpha-synuclein deposition in the same DLB patients would provide a more complete description of the mechanisms that underpin cognitive dysfunction in Lewy body diseases.

The present findings should be interpreted with several caveats. First, the modest sample size of our study could have obscured further group differences in [^18^F]-AV1451 binding, although it is comparable with previous studies (Gomperts et al., 2016; Kantarci et al., 2016; Smith et al., 2018). Moreover, we provide a transdiagnostic comparison between DLB and AD that yielded similar findings to AUC documented in previous studies (Kantarci et al., 2016; Ossenkoppele et al., 2018). Nevertheless, the sample size may hinder the generalizability of our AUC analyses of the medial temporal lobe meta-ROI and should be replicated in further studies to establish the clinical utility for differential diagnosis between DLB and AD. We also refrained from a fine-grained voxel-wise comparison of [18F]-AV1451 due to the small sample. In light of elevated [^18^F]-AV145 being reported in focal regions (Coughlin et al., 2020), it is possible that these changes could be obscured by our ROI approach. Second, only a subset of our patients with DLB had undergone [^11^C]-PiB imaging, precluding a robust investigation into the role of Aβ for tau propagation in DLB. A deeper characterization of the interactions between Aβ and tau on the trajectory of disease progression and clinical presentation should be a subject of further studies. Third, our DLB group was clinically defined and therefore autopsy confirmation is lacking, although the McKeith criteria adopted in this study has been shown to be specific for DLB pathology. Healthy controls were recruited on the basis of clinical assessment without biomarker confirmation. Considering the frequent observation of elevated Aβ in older individuals that do not show overt signs of cognitive impairment, age and Aβ-related tau deposition may have reduced the sensitivity of our group comparisons. Finally, the neuropathologic substrates of [^18^F]-AV1451 binding are still not fully understood in DLB, and further studies to evaluate the agreement between antemortem [^18^F]-AV1451 imaging and autopsy measurements of NFTs are needed (Coughlin et al., 2020; Lowe et al., 2016, 2020; Truocchio et al., 2020).

## 5 Conclusions

Our findings of (i) minimal tau binding in DLB and (ii) its associations with cognitive impairment highlight that tau imaging may have clinical relevance in the prognosis of DLB. While the mechanisms through which low tau burden may underlie cognitive impairment is still unclear, our preliminary demonstration of colocalization between [^18^F]-AV1451 and [^11^C]-PK11195 suggests that tau may have synergistic interactions with neuroinflammation and other processes in DLB. Tau imaging may also have translational potential to aid in the differential diagnosis of AD and DLB. On the basis of elevated [^18^F]-AV1451 binding in the medial temporal lobe, tau imaging could also be used to improve the identification and enrollment of probable patients with DLB into clinical trials to assess the therapeutic potential of antitau interventions. Further studies are necessary to validate our findings from longitudinal cohorts.

## Disclosure statement

John T. O'Brien has no conflicts related to this study. Unrelated to this work he has received honoraria for work as DSMB chair or member for tauRx, Axon, Eisai, has acted as a consultant for Roche, and has received research support from Alliance Medical and Merck. James B Rowe serves as an associate editor to Brain and is a nonremunerated trustee of the Guarantors of Brain and the PSP Association (UK). He provides consultancy to Asceneuron, Biogen, and UCB and has research grants from AZ-Medimmune, Janssen, and Lilly as industry partners in the Dementias Platform UK.

The data contained in the manuscript being submitted have not been previously published, have not been submitted elsewhere, and will not be submitted elsewhere while under consideration at Neurobiology of Aging.

All authors have reviewed and approved the submission of the data.

## CRediT authorship contribution statement

**Elijah Mak:** Resources, Formal analysis, Writing - original draft. **Nicolas Nicastro:** Resources, Formal analysis, Writing - original draft. **Maura Malpetti:** Writing - review & editing. **George Savulich:** Writing - review & editing. **Ajenthan Surendranathan:** Writing - review & editing. **Negin Holland:** Writing - review & editing. **Luca Passamonti:** Writing - review & editing. **P Simon Jones:** Writing - review & editing. **Stephen F. Carter:** Writing - review & editing. **Li Su:** Writing - review & editing. **Young T. Hong:** Writing - original draft, Writing - review & editing. **Tim D. Fryer:** Writing - original draft, Writing - review & editing. **Guy B. Williams:** None. **Franklin Aigbirhio:** Funding acquisition, Supervision, Methodology, Writing - review & editing. **James B. Rowe:** Funding acquisition, Supervision, Methodology, Writing - review & editing. **John T. O'Brien:** Funding acquisition, Supervision, Methodology, Writing - review & editing.

## Appendix A Supplementary data

Supplementary Fig. 1 Regional distribution of [^18^F]-AV1451 binding (BP_ND_) in DLB, HC, and AD. Abbreviations: AD, Alzheimer's disease; DLB, dementia with Lewy bodies; HC, healthy controls.

Supplementary Fig. 2 Elevated [^18^F]-AV1451 binding (BP_ND_) in AD relative to DLB. Regions are presented after post hoc Tukey honest significance difference and FDR p < 0.05, adjusted for age. Abbreviations: AD, Alzheimer's disease; DLB, dementia with Lewy bodies; FDR, False Discovery Rate.

NBA_tender_submission_supplementry
