# Supplementary figures and images for: Imaging tau burden in dementia with Lewy bodies using [18F]-AV1451 positron emission tomography
Source: Neurobiol Aging. 2021 May;101:172–80. doi: 10.1016/j.neurobiolaging.2020.11.006 (PMC8209140; doi:10.1016/j.neurobiolaging.2020.11.006)

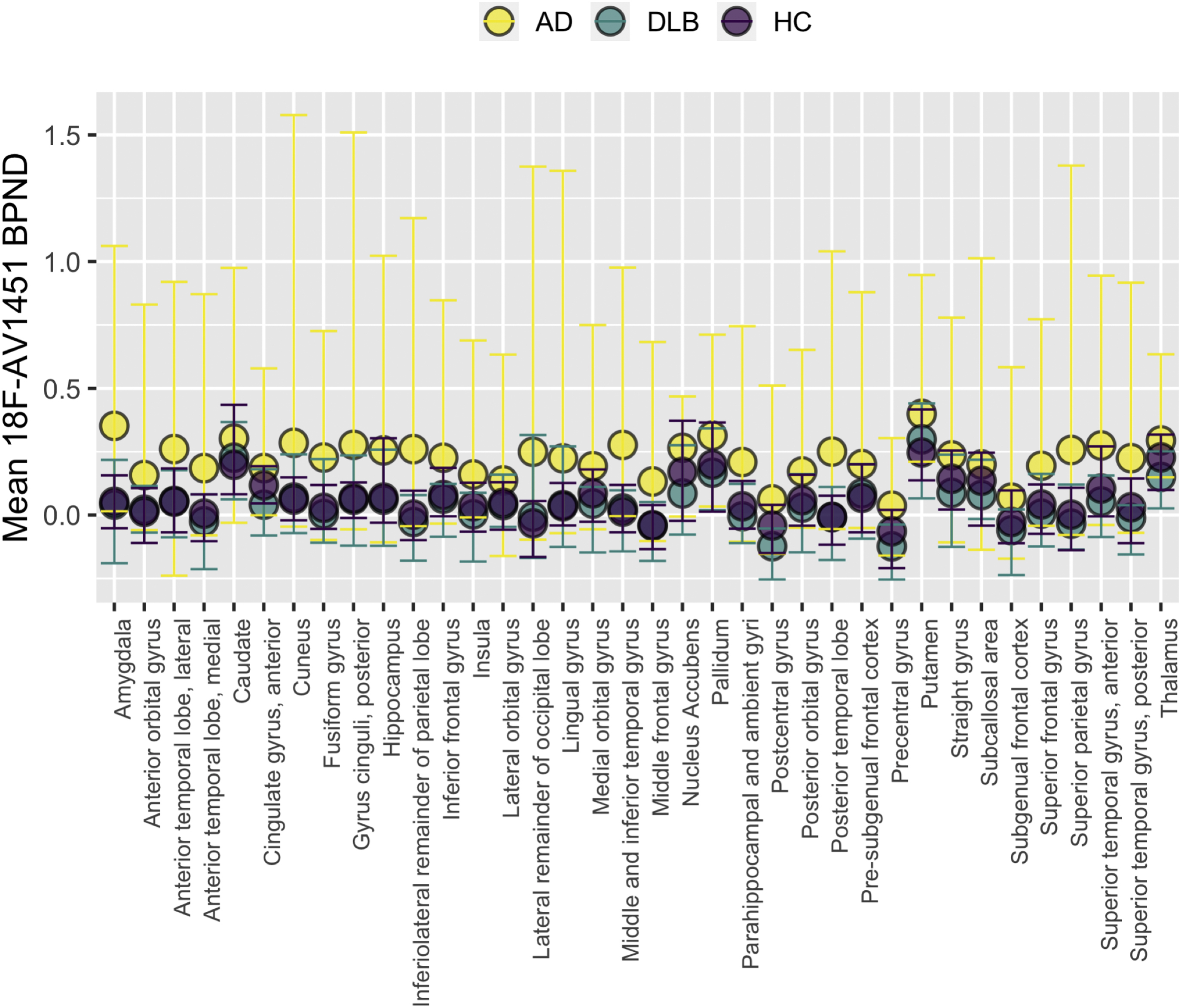

Supplement: Supplementary Fig. 1 — Regional distribution of [18F]-AV1451 binding (BPND) in DLB, HC, and AD. Abbreviations: AD, Alzheimer's disease; DLB, dementia with Lewy bodies; HC, healthy controls. [file figs1.jpg]

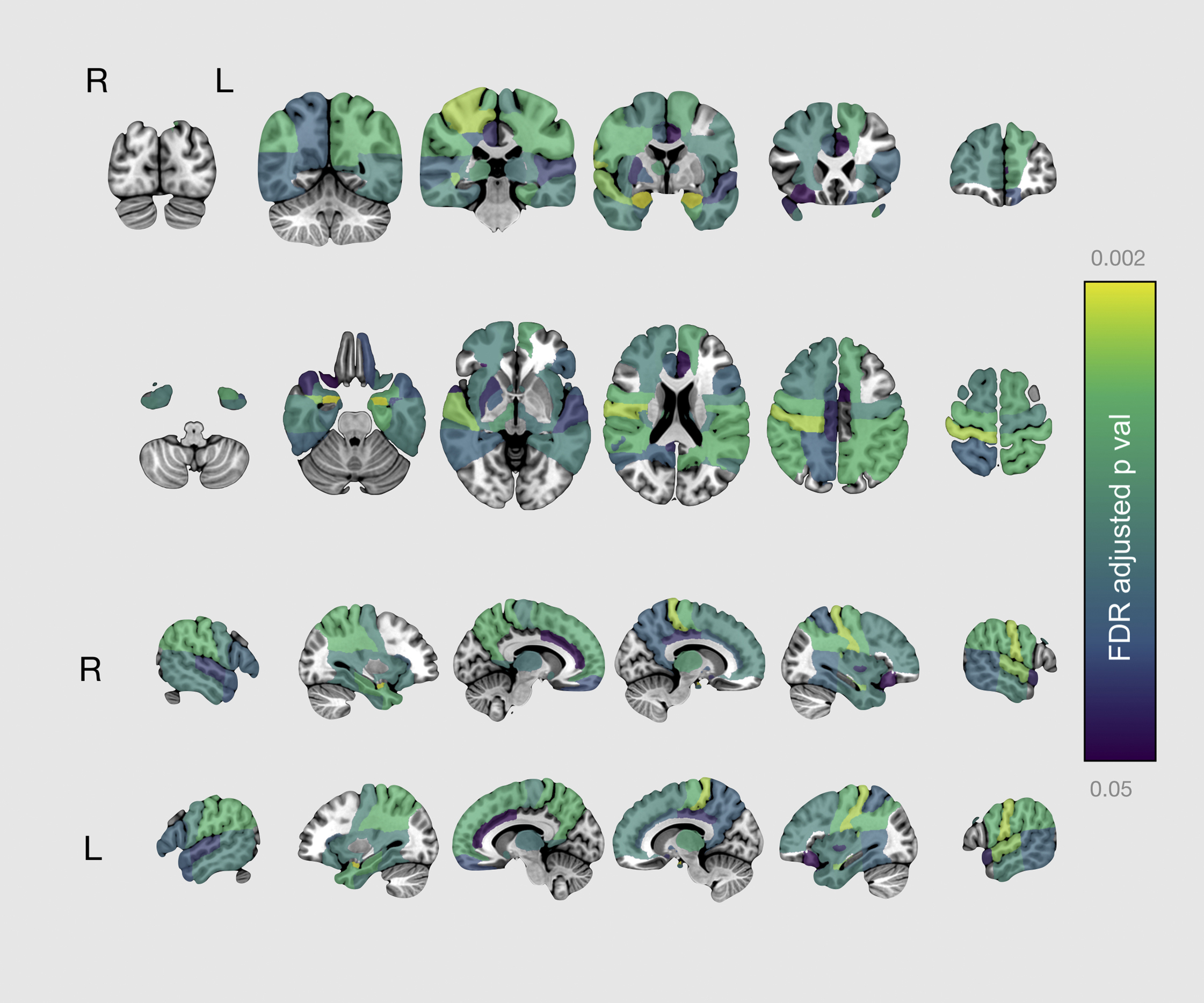

Supplement: Supplementary Fig. 2 — Elevated [18F]-AV1451 binding (BPND) in AD relative to DLB. Regions are presented after post hoc Tukey honest significance difference and FDR p < 0.05, adjusted for age. Abbreviations: AD, Alzheimer's disease; DLB, dementia with Lewy bodies; FDR, False Discovery Rate. [file figs2.jpg]
