# Supplementary material for: Imaging tau burden in dementia with Lewy bodies using [18F]-AV1451 positron emission tomography
Source: Neurobiol Aging. 2021 May;101:172–80. doi: 10.1016/j.neurobiolaging.2020.11.006 (PMC8209140; doi:10.1016/j.neurobiolaging.2020.11.006)
Supplement: NBA_tender_submission_supplementry [file mmc1.docx]

**SUPPLEMENTARY MATERIAL**

We illustrated the correlation between the partial volume corrected and non-partial volume corrected [^18^F]-AV1451 BP_ND_ below, across the regions in the Hammers atlas and for each subject (represented by the slopes). As expected, the correlation was highly significant across all groups (refer to R values below). Within each region of the Hammers atlas, the spearman R ranged from 0.8 to 0.9 (total sample of controls, DLB and AD). An illustration of the regional R values, sorted from lowest to highest, is shown in the dot plot below.


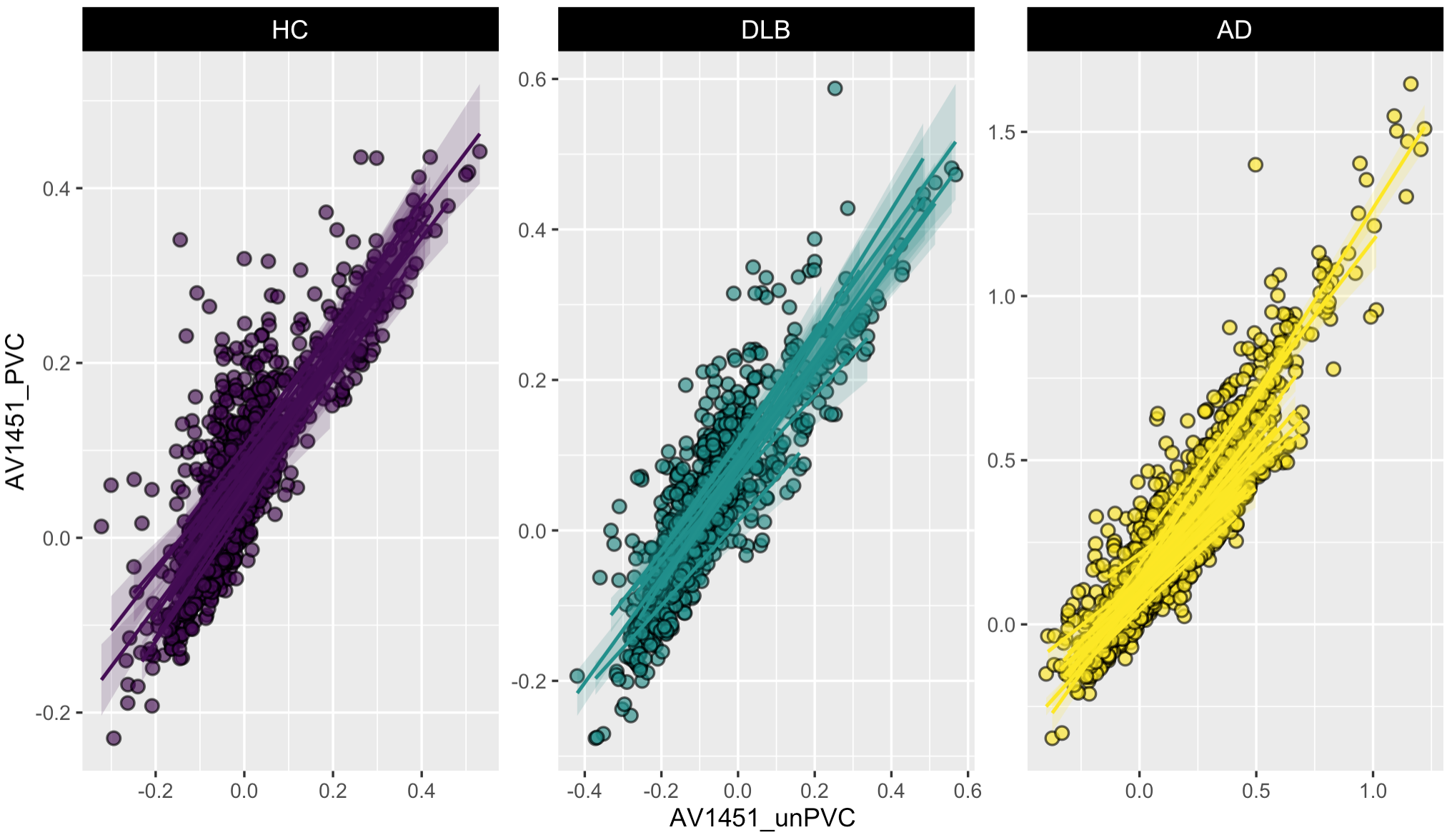


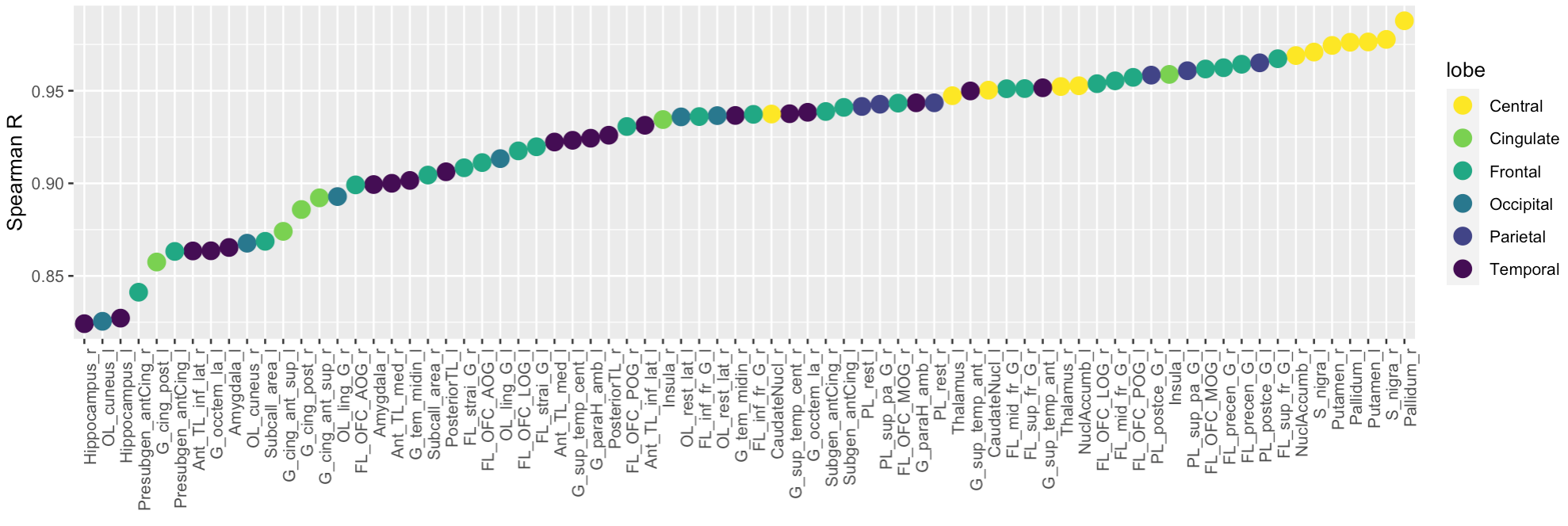


The effect of PVC on the AV1451 is illustrated via the density plots below, showing an expected increased recovery of [^18^F]-AV1451 signal in the DLB group relative to controls. Repeated analyses on the un-PVC datasets also did not change any of our key findings, although correlations with cognitive data were attenuated due to larger inter-variability of the non-PVC dataset. The decreased correlation between non-PVC [^18^F]-AV1451 with clinical measurements had also been documented in a previous study in a DLB cohort (Kantarci et al., 2016)


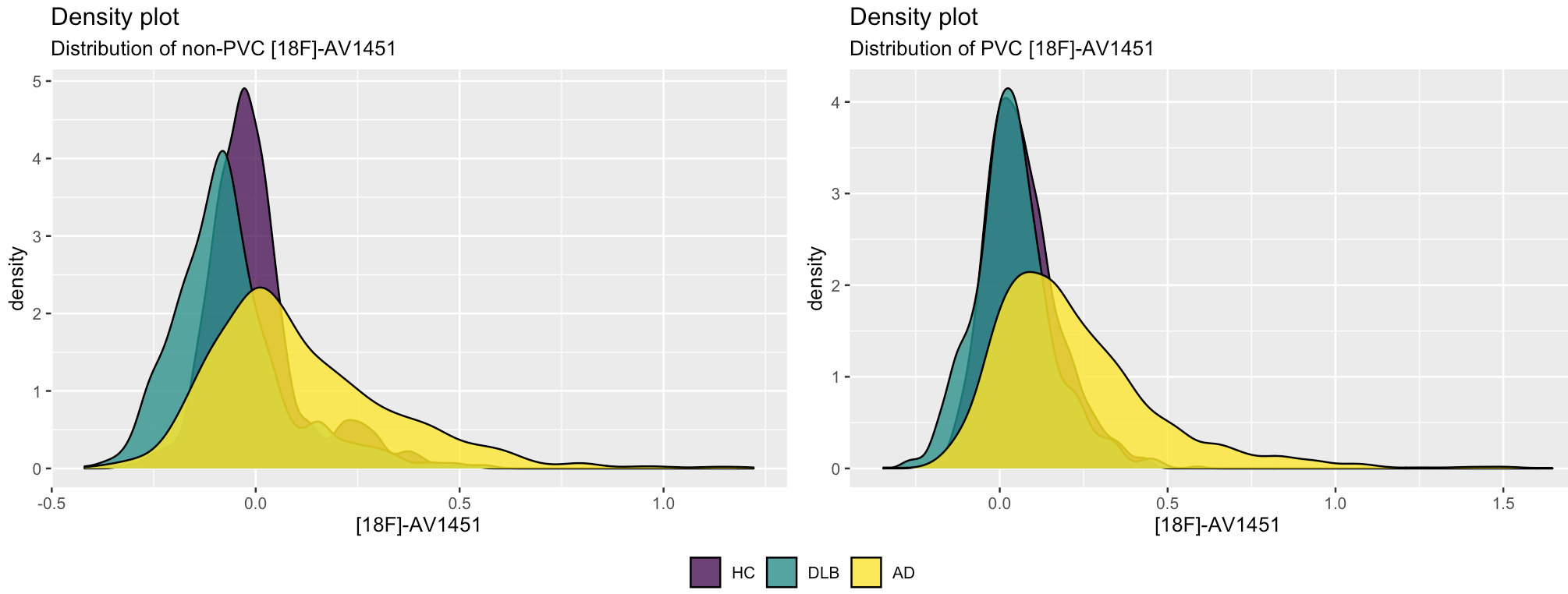


**REFERENCES**

Kantarci, K., Lowe, V.J., Boeve, B.F., Senjem, M.L., Tosakulwong, N., Lesnick, T.G., Spychalla, A.J., Gunter, J.L., Fields, J.A., Graff-Radford, J., Ferman, T.J., Jones, D.T., Murray, M.E., Knopman, D.S., Jack, C.R., Petersen, R.C., 2016. AV-1451 Tau and β-Amyloid PET Imaging in Dementia with Lewy Bodies. Ann. Neurol. 1–35. https://doi.org/10.1002/ana.24825
